# Book Notices

**Published:** 1885-02-20

**Authors:** 


### BOOKS AND PAMPHLETS RECEIVED.

*Transactions of the Medical Society of the State of Pennsylvania* at its thirty-fifth annual session, held at Philadelphia, May 14–16, 1884.

The above is a large, neatly gotten up work of over 600 pages, containing numerous able and interesting papers, with articles and reports from county societies, including lists of officers, members, etc., and a full index of the volume. We note, also, a list of all the presidents of the society from 1848 down to the present time.

The following are the present officers of the State Society, to wit: President, Dr. Henry H. Smith, Philadelphia. Vice Presidents, 1. Dr. E. Phillips, Schuylkill County ; 2. Dr. H. B. VanVabzah, Clairfield County ; 3. Dr. J. W. Kerr, York County ; 4. Dr. S. S. Shultz, Mountour County. Permanent Secretary W. B. Atkinson, M D., Philadelphia. Recording Secretary, M. S. French. Corresponding Secretary, J. G. Lee, M. D., Philadelphia.

The next meeting of the Society will be at Scranton on the last Wednesday in May next.

*The Diagnosis and Treatment of Chronic Nasal Catarrh.*

Three clinical lectures, delivered at the College of Physicians and Surgeons, New York, by George Norwood Lefferts, A. M., M. D., Professor of Laryngoscopy and Diseases of the Throat, in the College of Physicians and Surgeons ; consulting Laryngoscopic Surgeon to St. Luke's Hospital, and the New York Skin and Cancer Hospital, etc. Reprint from Medical News, Philadelphia, etc. St. Louis, Lambert & Co.

*A Comprehensive Anatomy, Physiology, and Hygiene, adapted for schools, academies, colleges, and families ;* containing brief directions for illustrative dissections of mammals ; for elementry work with the microscope ; for physiological demonstrations on the human body, and for the management of emergent cases. By John C. Cutter, B. S., M. D., Professor of Physiology and Comparative Anatomy in the Imperial College of Agriculture, Sapporo, Japan ; consulting Physician to Sapporo Ken Hospital, etc. ; with 150 illustrations. Philadelphia, J. B. Lippincott & Co. 1885.

The above work of 376 pages, neatly gotten up, and illustrated, though especially adapted to common schools, is admirably suited to first course students in physiology. It is plain, concise, and eminently practical, being easily comprehended even by the non-professional reader. To the general reader, and one seeking information without a teacher, it is, perhaps, better suited than any work of its class ; and the Glossary will be found of great advantage to readers of the classes named, and especially to beginners.

*The Basic Pathology and Pacific Treatment of Diphtheria, Typhoid, Zymotic, Septic, Scorbutic, and Putrescent Diseases generally.* By George J. Zeigler, M. D., late Physician to the Philadelphia Hospital ; Member of the Philadelphia County Medical Society ; of the American Medical Association ; author of "Zoo Adynamia," "Researches on Nitrous Oxide," "Natural Laws of Marriage," etc. Philadelphia : George J. Zeigler, M. D. 1880. Price, $2.

This work contains 225 pages, and is interesting in that the author holds that all the varied and complex diseases classed as scorbutic, necræmic, typhoid, zymotic, septic, infectious, malarial, gangrenous, putrescent, and allied affections have a common alkaline origin—ammonia, etc. The treatment suggested is in accordance with this theory. The work will repay perusal.

*Modern Medical Therapeutics :* a Compendium of Recent Formulæ and Specific Therapeutical Directions. By George H. Napheys, A. M., M. D. Edited by Joseph F. Edwards, M. D., and D. G. Brinton, M. D. Eighth edition, enlarged and revised. 8vo, pp. 629. Price, cloth, $4 ; sheep, $5. Philadelphia, D. G. Brinton, 1885.

Of the thoroughly useful books to the practicing physician, there is, probably, none to which he would refer oftener and with greater satisfaction than the one above named. It has stood the test of seven editions, and now appears for the eighth time, bringing the art of applied therapeutics down to the close of 1884, The revision it has undergone has been a most searching one, as the numerous omissions from previous editions testify. But the additions have been greater. In spite of dropping many procedures and articles which have not stood the test of experience well enough to justify their retention, and in spite of relegating the section on Diseases of Children to another volume, the present edition is noticeably bulkier than those which preceded it. The new matter, indeed, amounts to about one hundred and fifty pages.

*A Manual of Bandaging, adapted for Self-instruction.* By C. Henri Leonard, A. M., M. D., Professor of the Medical and Surgical Diseases of Women and Clinical Gynecology, Michigan College of Medicine; Member of the American Medical Association; of the Michigan State Medical Society; of the Wayne County Medical Society; and honorary member of the Canada Medical Association. With 139 engravings. Second edition, revised and enlarged. Published by the International Medical Journal Company. Detroit. Price, $1.50.

We regard this a very practical and useful work, both to the student and practitioner.

*The Story of My Life*, by J. Marion Sims, M.D.; New York: D. Appleton & Co., No. 5, Bond Street.

This is an Autobiography of Marion Sims gotten out by the son since the death of the honored father. It must prove a work of great interest to the medical profession throughout the world. It contains 471 pages, and is neatly gotten out in cloth.

*One Hundred Years of Publishing*, 1785—1885; Philadelphia: Lea Brothers & Co., 1885.

A beautiful little work, gotten up in most elegant typographical taste, containing a history of that famous Publishing House, Lea Brothers, the latest successors of those who preceded them in the last one hundred years.

We warmly appreciate the card and compliments sent us by this old and worthy establishment.

*Physicians' Daily Pocket Record*, comprising a visiting list, many useful Memoranda, Tables, etc. Nineteenth Year—New and Revised, with Metric Posological Table, etc. By D. G. Brinton, M.D. Philadelphia: Office of Medical and Surgical Reporter, 115 S. 7th St.

*School Hygiene in Relation to its Influence upon the Vision of Children, or School Sanitation.* An address delivered before the Medical Association of Georgia, 1884, by A. W Calhoun, M.D., President, Atlanta, Ga.

*Treatment of the Insane*, by Orphêus Everts, M D., Medical Superintendent of the Cincinnati Sanitarium, Vice President Association of American Superintendents of Insane Hospitals, &c., &c.

*Sixth Annual Report of the Board of Health of Augusta, Georgia.*

*Eleventh Annual Report of the Superintendent of the Cincinnati Sanitarium*, for the year ending November 30th, 1884.

*Acetate of Lead in Ocular Therapeutics*, by David DeBeck, M.D., Assistant to the Chair of Ophthalmology.

